# The processivity factor Pol32 mediates nuclear localization of DNA polymerase delta and prevents chromosomal fragile site formation in Drosophila development

**DOI:** 10.1371/journal.pgen.1008169

**Published:** 2019-05-17

**Authors:** Jingyun Ji, Xiaona Tang, Wen Hu, Keith A. Maggert, Yikang S. Rong

**Affiliations:** 1 School of Life Sciences, Sun Yat-sen University, Guangzhou, China; 2 Laboratory of Biochemistry and Molecular Biology, National Cancer Institute, National Institutes of Health, Bethesda, Maryland, United States of America; 3 Department of Cellular and Molecular Medicine, University of Arizona, Tucson, AZ, United States of America; Geisel School of Medicine at Dartmouth, UNITED STATES

## Abstract

The Pol32 protein is one of the universal subunits of DNA polymerase δ (Pol δ), which is responsible for genome replication in eukaryotic cells. Although the role of Pol32 in DNA repair has been well-characterized, its exact function in genome replication remains obscure as studies in single cell systems have not established an essential role for Pol32 in the process. Here we characterize Pol32 in the context of *Drosophila melanogaster* development. In the rapidly dividing embryonic cells, loss of Pol32 halts genome replication as it specifically disrupts Pol δ localization to the nucleus. This function of Pol32 in facilitating the nuclear import of Pol δ would be similar to that of accessory subunits of DNA polymerases from mammalian Herpes viruses. In post-embryonic cells, loss of Pol32 reveals mitotic fragile sites in the Drosophila genome, a defect more consistent with Pol32’s role as a polymerase processivity factor. Interestingly, these fragile sites do not favor repetitive sequences in heterochromatin, with the *rDNA* locus being a striking exception. Our study uncovers a possibly universal function for DNA polymerase ancillary factors and establishes a powerful system for the study of chromosomal fragile sites in a non-mammalian organism.

## Author summary

Cancer etiological studies suggest that the majority of pathological mutations occurred under near normal DNA replication conditions, emphasizing the importance of understanding replication regulation under non-lethal conditions. To gain such a better understanding, we investigated the function of Pol32, a conserved ancillary subunit of the essential DNA polymerase Delta complex, through the development of the fruit fly Drosophila. We uncovered a previously unappreciated function of Pol32 in regulating the nuclear import of the polymerase complex, and this function is developmentally regulated. By utilizing mutations in *pol32* and other replication factors, we have started to define basic features of Chromosome Fragile Sites (CFS) in Drosophila somatic cells. CFS is a major source of genome instability associated with replication stresses, and has been an important topic of cancer biology. We discovered that CFS formation does not favor genomic regions with repetitive sequences except the highly transcribed locus encoding ribosomal RNA. Our work lays the groundwork for future studies using Drosophila as an alternative system to uncover the most fundamental features of CFS.

## Introduction

Genome replication is of paramount importance to life. Although we have ample understanding of the biochemistry of DNA replication at the molecular level, the complexity of replication regulation is much less understood. In particular, the functions of proteins deemed “ancillary factors” are less understood than those of the catalytic components of the DNA replication machinery. The importance of understanding the functions of these factors is highlighted by the remarkable finding that the yeast Pol δ catalytic enzyme can be functionally replaced *in vivo* by a viral polymerase provided its C-terminal domain retains efficient interactions with ancillary replication factors [1]. Understanding such regulatory roles is also important for improving human health, as while a loss of replication capacity is often lethal, defective regulation might be more compatible with various disease states including cancer. The importance of studying cellular responses to non-lethal perturbation of DNA replication (or replication stress) is further emphasized by the results from recent cancer etiological studies suggesting that the majority of pathological mutations likely occurred under normal or near normal DNA replication conditions [2, 3].

One of the consequences of perturbing replication is the formation of chromosomal fragile sites [reviewed in 4]. These fragile sites appear as visible gaps or constrictions on mitotic chromosomes formed under replication stresses, and can be a source of genome instability by, e.g., initiating aberrant recombination. Mammalian studies have uncovered detailed features of chromosome fragile sites. Many genomic regions generally considered “hard-to-replicate”, such as repetitive sequences with a tendency to form secondary structures, are more sensitive to replication stress [e.g. 5–7]. Several large genes with complex transcription and replication patterns are common fragile sites in mammals [reviewed in 8, 9]. The extent to which the features of mammalian fragile sites are conserved through evolution remains unclear, as is the case for common molecular characteristics of fragile sites in yeast [10]. Therefore, more experimental systems are needed for the study of fragile sites to uncover their most fundamentally conserved mechanistic and phenomenological characteristics.

DNA polymerase δ is one of the major genome-replicating machineries in eukaryotes. Its subunit composition is highly-conserved from yeast to mammals, including minimally the catalytic subunit PolD, a “structural” subunit of Pol31 and an “ancillary” subunit of Pol32 [for reviews on DNA pol δ, see 11–13]. Although Pol32 is biochemically defined as a processivity factor of Pol δ, ensuring maximal DNA synthesis efficiency of the complex [14–16], the necessity for Pol32 in genome replication could not be established in multiple organisms. Contrary to *pol3* (*S*. *cerevisiae polD*) or *pol31*, deletion of *pol32* is not lethal in budding yeast, although *pol32* mutants are sensitive to exogenously applied replication stresses [17]. In fission yeast, deletion of *cdc27* (*S*. *pombe pol32*) is lethal due to defective chromosome segregation but nonetheless does not lead to gross defects in genome replication [18, 19]. More strikingly, chicken DT40 cells homozygous for a *polD3* deletion are viable and exhibit a normal cell cycle profile (PolD3 and p66 are names given to Pol32 homologs in higher eukaryotes) [20]. Furthermore, human cells can sustain a significant knockdown of PolD3 level without apparent effects on DNA replication [21, 22]. However, mouse knockout mutations of *polD3* were shown to cause embryonic lethality, and conditional reduction of *polD3* in adult B cells and embryonic stem cells caused defects in BrdU incorporation and cell cycle progression [23, 24]. As no clearly consistent trend is discernible, results from all these studies suggest that a comprehensive understanding of Pol32 needs to involve an analysis of cell type and developmental stage in dissection of its function.

A role for Drosophila Pol32 in DNA repair has been previously characterized [25], and another study revealed that Pol32 is essential for the prevention of chromosome breakage in proliferating cells [26]. Whether Pol32 is required for genome replication has not been investigated in Drosophila. Here we showed that Pol32 is absolutely required for genome replication during the earliest cell cycles, and this function is endowed by Pol32’s ability to facilitate nuclear localization of the Pol δ complex. However, in post-embryonic cells, loss of Pol32 does not block genome replication but instead sensitizes cells to the formation of chromosomal fragile sites. Although a significant portion of the breaks happen in regions enriched with repetitive sequences, these regions are not particularly favored for fragile site formation, with the *rDNA* locus being the one clear exception.

## Results

### Loss of maternal Pol32 abolishes genome replication in syncytial embryos

An important role of Pol32 in DNA double strand break (DSB) repair in Drosophila was established using mutant alleles that we generated [25]. What we also observed but did not report in depth at the time, was somatic phenotypes of *pol32* homozygotes. Mutant adults express a variable degree of bristle loss or shortening (S1 Fig), and females are sterile while males are fertile. These somatic phenotypes are similar to those reported in a previous study of *pol32* in Drosophila [26].

**Fig 1:**
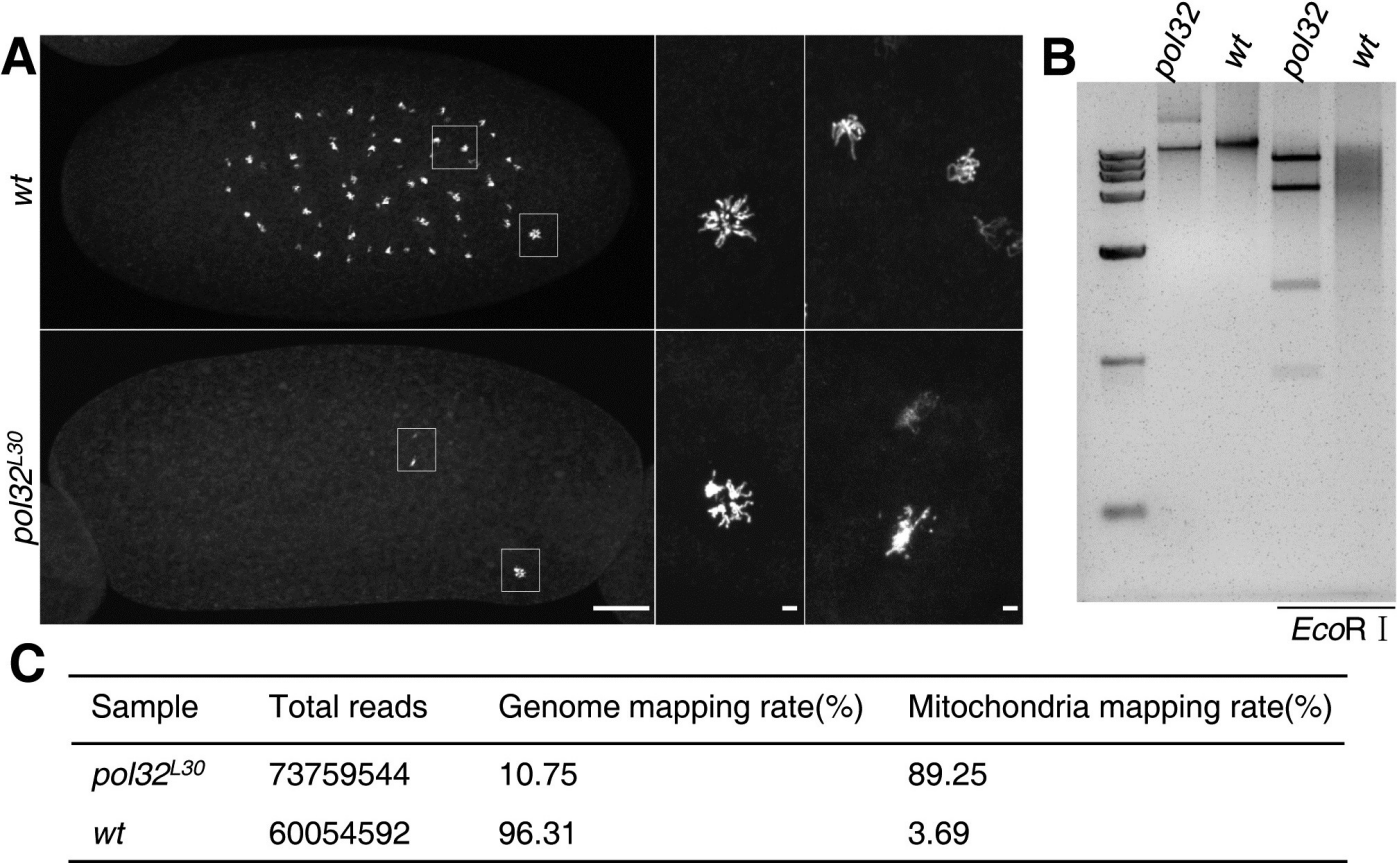
Loss of Pol32 impedes genomic replication. **A**. DAPI stained embryos of wild-type and *pol32* mutants. These are embryos collected at 0-2hr after egg laying. Two areas of enlargement marked with rectangles are shown in the middle and the right panels, with the middle ones showing the polar bodies and the right ones showing zygotic nuclei. **B**. Agarose gel showing total embryonic DNA from mutant (*pol32*) and wild-type (*wt*) embryos either uncut (left two lanes) or cut with *Eco*RI (right two lanes). The markers in the leftmost lane, from top to bottom, have sizes in kb of: 15, 10, 7.5, 5, 2.5, 1, 0.25. **C**. Summary results from whole genome sequencing showing DNA from *pol32* mutant embryos primarily consists of mitochondrial DNA. Scale bars indicate 40μm.

We noticed that *pol32* homozygous females lay eggs that do not hatch. We hereafter refer these as *pol32*-mutant embryos even though they are genotypically heterozygous as they had wild type fathers. To assess embryonic development of *pol32*-mutants, we DAPI-stained whole-mount embryos and discovered that they were fertilized but that no embryo (N>1000) had more than 8 foci of DAPI-bright material (Fig 1A), indicating that these embryos were arrested very early in development. In Drosophila, the first 13 cell cycles rely solely on maternally supplied protein and RNA molecules. Loss of Pol32 causes maternal effect lethality, which suggests that the presence of maternally deposited Pol32 protein is essential for embryonic development, possibly by ensuring genome replication.

We isolated total DNA from 0–2 hr embryos collected from either wild-type or mutant females, digested the two samples with *Eco*RI, and electrophoretically separated them on an agarose gel. The DNA sample from the mutants revealed a distinct set of four bands, an interesting pattern that is different from the smeary appearance of digested wild-type DNA (Fig 1B). This pattern of *Eco*RI digestion is consistent with that the DNA extracted from mutants consisting mostly of mitochondrial DNA, based on known sequence of the Drosophila mitochondrial genome [27]. To confirm this hypothesis, we subjected these DNA samples to whole genome sequencing, and found that about 90% of the reads from the mutant sample are mapped to the mitochondrial genome, whereas that number was less than 4% for DNA extracted from the wild-type sample (Fig 1C). We therefore conclude that there is very limited genome replication in *pol32*-mutant embryos, strongly suggesting that Pol32 is critically required for nuclear replication during early development.

### Cellular localization of Pol32 during development

We generated an antibody against Drosophila Pol32. This antibody recognizes a protein band on Western blot from wild-type but not *pol32* mutant tissues (S2A Fig) confirming its specificity, although the observed size of Pol32 protein is around 65 KD, larger than the predicted size of 47 KD. Interestingly, the mammalian Pol32 homolog PolD3/p66 has an estimated size of 66 KD on an SDS-PAGE gel, also larger than the predicted size of 51 KD [28], and *S*. *pombe* Cdc27 migrates slower than the expected size [15]. The cause for this common behavior of Pol32 proteins is not known.

**Fig 2:**
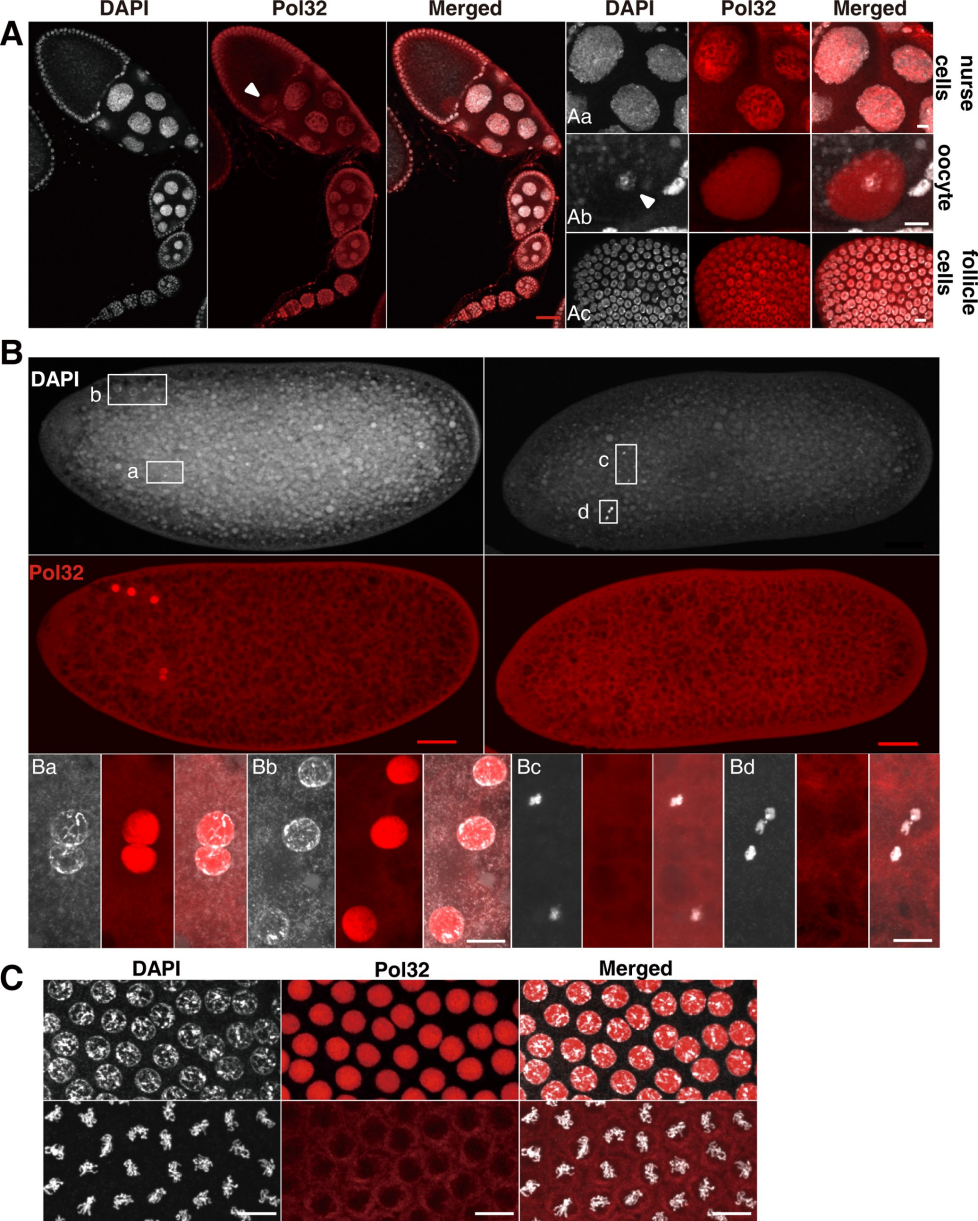
Pol32 localization during normal development. A separate image is provided for the DAPI signal (in white), the anti-Pol32 signal (in red), and the merged product of the two channels. **A**. Pol32 in ovarian tissue. A series of egg chambers (left three panels) are shown with three enlarged areas included in the right three columns of images. The nucleus of the oocyte is marked with an arrowhead. In **Aa**, Pol32 can be seen in the nuclei of nurse cells. In **Ab**, Pol32 can be seen in the nucleoplasm of the oocyte with chromosomes marked with an arrowhead. In **Ac**, Pol32 can be seen in nuclei of follicle cells. **B**. Pol32 in 0-2hr old embryos. The top left panels show images of an embryo in interphase, and the top right panels showing an embryo with condensed chromosomes. The five haploid nuclei (three polar body nuclei and the parental pronuclei) are shown underneath as enlarged images for each embryo. **Ba** and **Bb** show interphase nuclei. **Bc** and **Bd** show metaphase nuclei. **C**. Pol32 in syncytial cell cycles. The top row shows interphase cells with nuclear Pol32 signals. The bottom row shows mitotic cells with no Pol32 in the nucleus. Scale bars in red indicate 40μm, and 10μm in white.

To better understand the developmental regulation of Pol32, we performed immunostaining on some of the replicating tissues using this antibody. During oogenesis, Pol32 is ubiquitously present in the nucleus. In particular, polyploid nurse cells have abundant Pol32 (Figs 2A and S2B). Pol32 is also present in the nuclear space of the oocyte (Fig 2A), confirming that Pol32 is maternally deposited.

**Fig 3:**
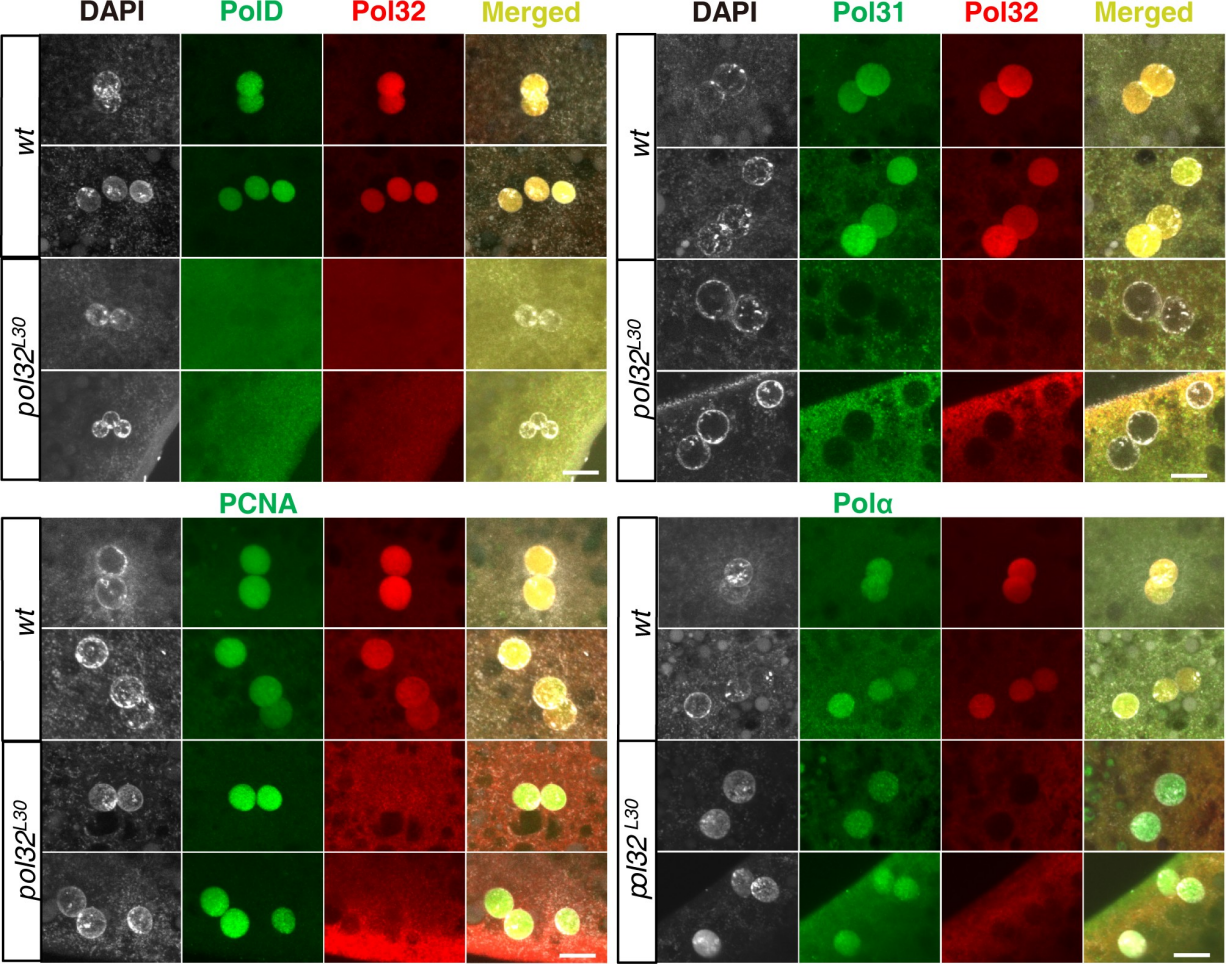
Pol δ complex is absent in embryonic nuclei of *pol32* mutants. Representative confocal images of antibody staining of the Pol δ complex (PolD, Pol31 and Pol32), PCNA and Polα in wild-type and *pol32*-mutant 0-2hr old embryos. For each antibody, 50 wild-type embryos were imaged. The number of *pol32*-mutant embryos imaged for each antibody are: 70 for PolD, 71 for Pol31, 52 for PCNA, and 59 for Polα. Scale bars indicate 10μm.

Since the *pol32* mutant phenotype manifests most strongly during early development, we next focused our study of Pol32 localization on early embryos. During the very first cell cycle when the male and female pronuclei fuse, Pol32 was associated with the parental nuclei as well as the three nuclei that eventually give rise to the polar body (Figs 2B and 3). Mutant embryos showed no anti-Pol32 signals (Fig 3), again confirming the specificity of our antibodies. Interestingly, wild-type nuclei with condensed chromosomes lack Pol32 signal, suggesting that Pol32 accumulation is associated with ongoing genome replication (Fig 2B). Consistent with this, Pol32’s nuclear localization is phasic during the later cell cycles in the embryo. Pol32 accumulates in the nucleus during interphase but disperses into the cytoplasm at the onset of mitosis (Fig 2C). We cannot exclude the possibility that some of the Pol32 is degraded during the mitotic program such that the level of Pol32 protein further fluctuates throughout the cell cycle. Since Pol32 is a subunit of the Pol δ enzyme complex, we were interested in the localization of the other subunits. We generated antibodies against PolD and Pol31, and observed a very similar localization pattern to that of Pol32 in the early embryonic cycles (S3 Fig). Thus, the cellular localization of multiple components of the Pol δ complex is consistent with its molecular role in genome replication.

### Pol32 is specifically required for nuclear localization of Pol δ in early embryos

**Fig 4:**
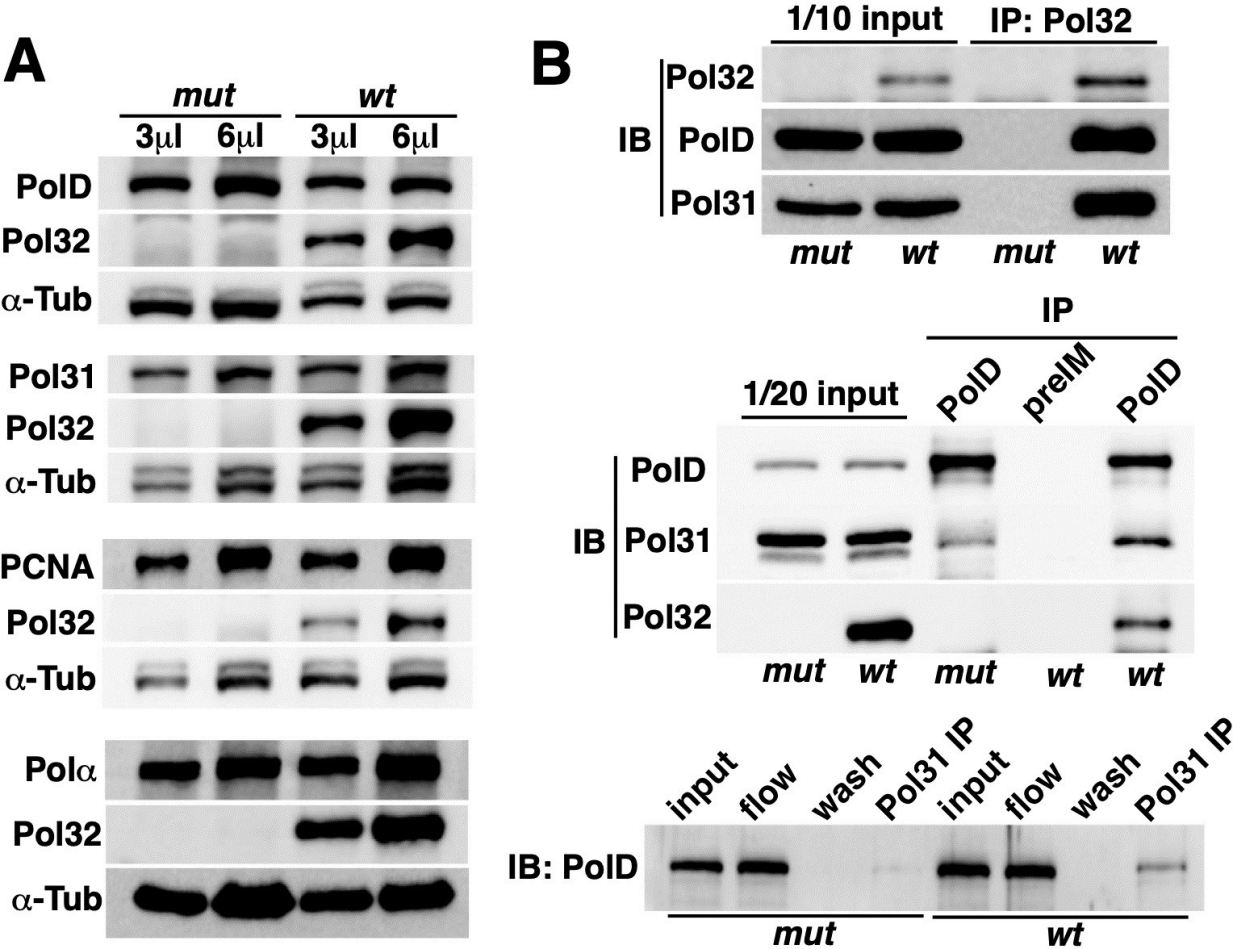
Interactions within the Pol δ complex. **A**. The level of Pol δ in wild-type and *pol32* mutant embryonic extracts. Different amount of extracts (3 or 6μl) were used for Western blot analyses with Tubulin as a general loading control. Genotypes are on top and antibodies are listed to the left of the images. **B**. Co-IP experiments to detect interactions among Pol δ subunits. For each panel, the genotypes are indicated at the bottom, and the antibodies used for IP on top of the images. “preIM” indicates that the corresponding pre-Immune serum was used for IP. The antibodies used for Western blot detection (IB) are listed to the left of the panels. In the bottom panel, “input” marks the lane with 1/20 of the input extract loaded. “flow” marks the lane with a sample of “flow through” in the IP experiments loaded. “wash” denotes the lane with a sample of “wash” in the IP experiments.

The lack of genome replication in *pol32*-mutant embryos, produced by *pol32* homozygous mothers, is in sharp contrast to the survival of *pol32* homozygous animals. We set out to better understand the underlying cause for embryonic lethality by localizing protein factors known to participate in genome replication with antibodies. As *pol32*-mutant embryos arrest very early in development, we focused our attention on the gonometric first zygotic division at the time when the juxtaposing parental pronuclei and the three polar body nuclei are undergoing replication. As shown in Figs 2B and 3, Pol32 is abundantly present in those five haploid nuclei. As expected, both PolD and Pol31 are also present (Fig 3). Remarkably, neither is present at similarly staged nuclei in *pol32*-mutant embryos (Fig 3). We first ruled out that this lack of localization was due to the absence of the proteins of interest in the embryos. In the Western blots shown in Fig 4A, both maternal PolD and Pol31 are present at a similar level to those in wild-type embryos. Secondly, we determined that this localization defect induced by the loss of Pol32 is specific to Pol δ as the localization of PCNA, a replication factor interacting with all three subunits of Pol δ in yeast [19, 29], was not affected (Fig 3). Moreover, the localization of the catalytic subunit of DNA polymerase α (Polα), which is required for initiating genome replication [30], was not affected by the lack of maternal Pol32 either (Fig 3). Therefore, loss of maternal Pol32 specifically inhibits the nuclear localization of the Pol δ complex.

**Fig 5:**
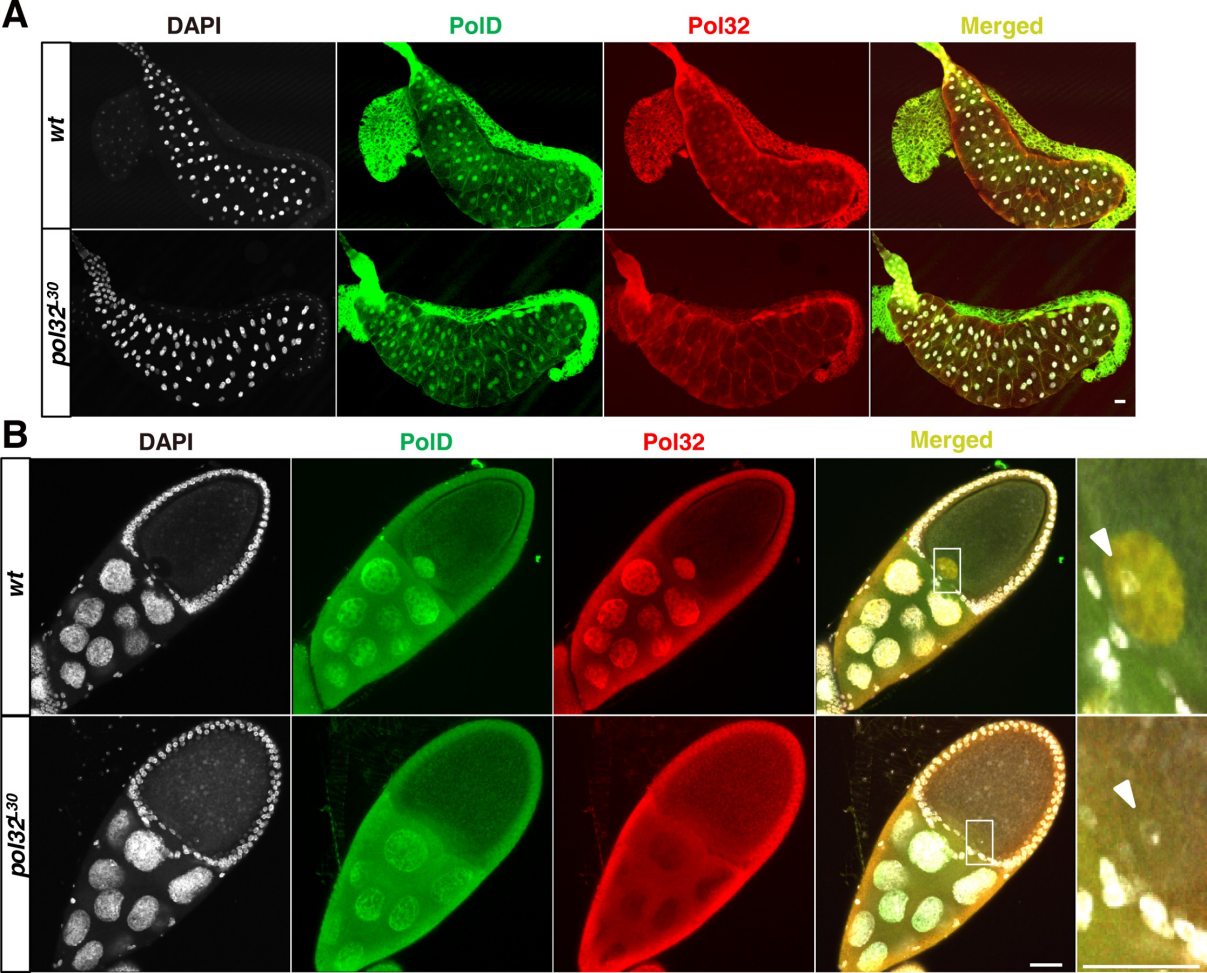
PolD remains nuclear localized in post-embryonic cells in *pol32* mutants. Confocal images showing PolD and Pol32 localizations in a larval salivary gland (**A**) and an egg chamber from adult ovary (**B**). The genotypes are indicated to the left. In the “merged” image in **B**, the nucleus of the oocyte is demarcated with a rectangular box, which is shown as enlarged images to the right with chromosomes marked with an arrowhead. Note the lack of PolD signals in the mutant. Scale bars indicate 40μm.

We also investigated the effect of *pol32* mutations on PolD location in post-embryonic cells. We have chosen polytene cells in the salivary glands of third instar larvae and cells in the adult ovary for our investigation as PolD is present in these cells under normal conditions (Fig 5). In *pol32* mutants, PolD is also present in the nucleus (Fig 5), which is expected since *pol32* homozygous mutants are viable with largely normal development. Interestingly, PolD is prominently missing from the nucleus of the mutant oocyte (enlarged image in Fig 5B), reminiscent of the situation in early embryos (Fig 3). This observation further strengthens our conclusion that the nuclear localization of the maternal PolD complex requires Pol32.

### The wHTH domain of Pol32 is functionally critical

To facilitate the identification of specific Pol32 domains required for its function, we set out to investigate the physical interactions among Pol δ subunits by immunoprecipitation (IP) using extracts from early embryos. As shown in Fig 4B, we detected interactions between Pol32 and PolD, Pol32 and Pol31, and PolD and Pol31, consistent with a hetero-trimeric complex. Interestingly, in the absence of Pol32, Pol31 remains capable of interacting with PolD (Fig 4B).

**Fig 6:**
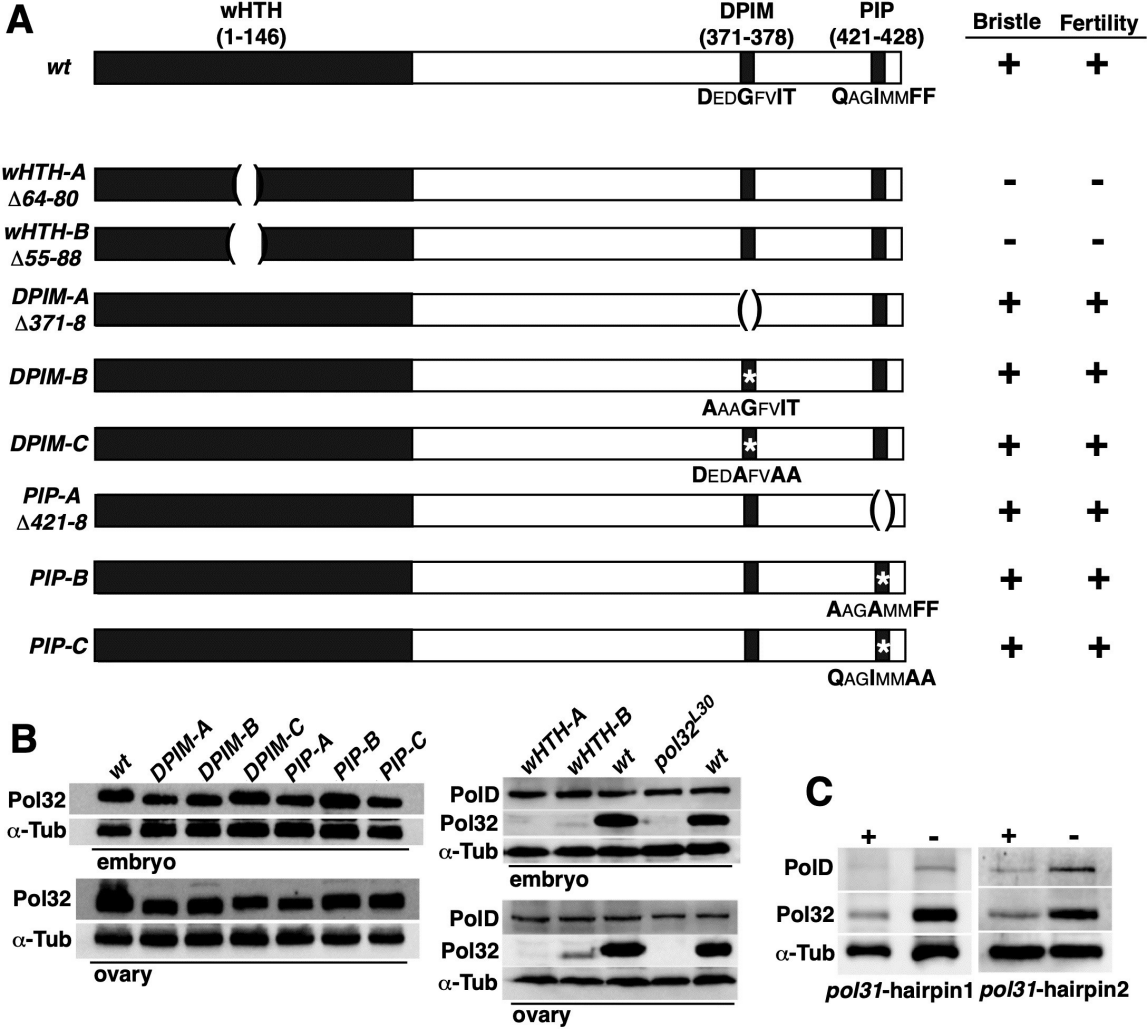
Pol32-Pol31 interaction is essential for Pol32 function. **A**. Constructs used in rescuing experiments and their effects on the mutant phenotypes. The 431aa Pol32 protein is denoted as a rectangular box with domains of interest labelled in black. The names of the domain are on top of the boxes with the range in amino acids in parentheses. For the Polα-interacting DPIM and the PCNA-interacting PIP domain, the amino acid sequences of the domain are listed underneath with the conserved residues in Bold and a larger Font. For constructs with a deletion, the deleted range in amino acids is shown under the name of the mutant construct and proceeded by a “Δ”. For constructs with residues changed to Alanine, the mutant composition of the mutated domain is shown underneath and the mutated domain denoted with an “*”. The constructs were tested for their abilities to rescue two mutant phenotypes. “Bristle” indicates shorten or missing bristles in *pol32* adults. “Fertility” indicates female sterility of *pol32* mutants. “+” indicates that a construct can recue the phenotype when introduced into a *pol32* mutant background, while “-” cannot. **B**. The levels of Pol32 mutant proteins. Western blots using total extracts from either ovaries or embryos were probed with antibodies indicated to the left of the images. The animals are *pol32* homozygotes with a rescuing construct listed on top of the images and shown in **A**. Tubulin was used as a general loading control. For the two *wHTH* mutants, the level of PolD was also measured (right panels in **B**). **C**. The effect of Pol31 RNAi on the level of Pol δ. Two transgenes carrying different RNAi hairpins were used to knockdown Pol31 in larval tissues. The levels of Pol32 and PolD were assayed by Western blotting. “+” indicates samples from animals with the hairpin construct, while “-” from control animals without the construct.

To identify specific protein interactions that might be responsible for facilitating Pol δ localization we generated point mutations, individually disrupting three known protein domains of Pol32 that interact with other replication factors (Fig 6A). In yeast and mammals, the Pol31-interacting region has been mapped to a winged helix-turn-helix (wHTH) domain at the N terminus of Pol32 [31–33]; a Polα-interacting DPIM domain has been mapped to a C-terminal region [34], and lastly a PCNA-interacting PIP box has been mapped to the C-terminus of Pol32 [19].

We constructed a genomic fragment from the *pol32* locus and were able to rescue the phenotypes of the mutants using this gene fragment as a rescuing transgene. Starting with this construct, we introduced small deletions or residue changes to the three domains of interest (Fig 6A), and transformed these constructs individually into a *pol32* mutant background and tested the effects on the bristle and fertility phenotypes. As summarized in Fig 6A and shown in S1 Fig, all gene constructions except those disrupting wHTH were able to rescue both defects, while mutations of wHTH failed to rescue either. In embryos produced by females with the *wHTH* mutations, PolD protein remains at or near its normal level (Fig 6B). These results strongly suggest that the wHTH domain, important for Pol32-Pol31 interaction, is required for Pol32 function in both embryonic and post-embryonic somatic cells.

Interestingly, the wHTH-mutated Pol32 protein was produced at a greatly reduced level (Fig 6B), suggesting that the mutant protein is unstable possibly due to its inability to interact with Pol31. Consistent with this hypothesis, when we reduced Pol31 level in post-embryonic cells with RNAi (see Materials and Methods), we observed a concomitant reduction of the otherwise normal Pol32 protein (Fig 6C). Alternatively, the instability of the wHTH-mutated Pol32 protein could be due to the missing of a few residues critical for its stability, we are currently unable to distinguish between these two hypotheses.

### Loss of Pol32 reveals mitotic fragile sites in the Drosophila genome

**Fig 7:**
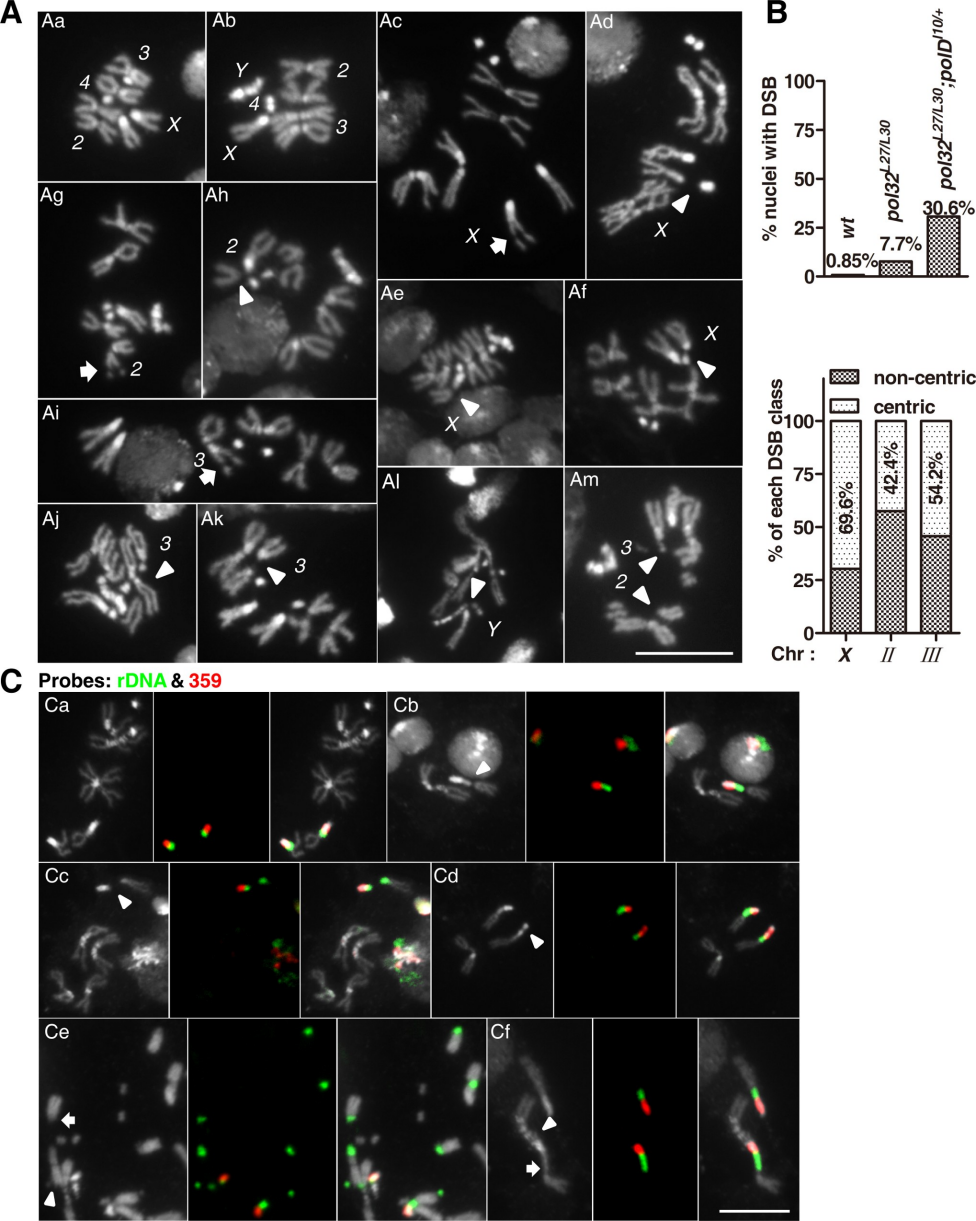
Loss of Pol32 induces chromosome breaks in post-embryonic cells. **A**. Mitotic figures showing the different classes of DSBs in the mutant *pol32*^*L27/L30*^*; polD*^*l10/+*^. In the two wild-type nuclei chromosomes are individually identified. In the mutant nuclei, only the chromosome with a DSB of interest is denoted and the approximate location of the DSB marked with either an arrowhead (centric DSB) or an arrow (non-centric DSB). **Aa, Ab**: two wild-type nuclei with no broken chromosomes. **Ac**: a non-centric *X* chromosome break. **Ad**: a DSB at the centromere-proximal *rDNA* region on *X*. **Ae**: a DSB at the distal *rDNA* region. **Af**: a DSB at the DAPI-bright block of *X*. **Ag**: a non-centric DSB on chromosome *2*. **Ah**: a centric DSB of *2*. Note the rest of chromosome *2* is missing in this nucleus. **Ai**: a non-centric DSB on chromosome *3*. **Aj, Ak**: two nuclei each with a centric DSB on *3*. **Al**; a DSB at the *rDNA* region of the *Y* chromosome. **Am**: multiple DSBs in a nucleus. **B**. DSB frequencies. The top chart quantifies the percentage of nuclei with at least one chromosome break in three different genetic backgrounds. The bottom chart quantifies the two classes of breaks on each major chromosome from neuroblasts of the genotype *pol32*^*L27/L30*^*; polD*^*l10/+*^, with the numbers indicating the percentages of centric DSBs. **C**. FISH identifies broken sites in *X* peri-centromeric region. In each triplet of images, to the left is a DAPI-stained chromosome figure; in the middle is FISH image of a nucleus double labelled with rDNA (green) and 359 satellite (red) probes; and to the right is the merged product. **Ca**, a normal female nucleus. **Cb**, a nucleus with a DSB at the distal region of *rDNA* where the sister chromatids remain synapsed. **Cc**, a nucleus with a DSB splitting the *rDNA* arrays into approximate halves. **Cd**, a nucleus with a DSB splitting the DAPI-bright 359 satellite arrays into approximate halves. **Ce**, severe *rDNA* instability. Numerous acentric chromosomal fragments are visible with *rDNA* at one end (arrow), and *rDNA* fragments are present that do not appear to be attached to any major chromosomes (arrowhead). **Cf**, a possible case of *rDNA* expansion. An *X* chromosome with a greatly expanded *rDNA* array (arrow), when compared with another *X* chromosome with a normal appearance. In the same nucleus, the 359 satellite is attached to an aberrant chromosome proximally (arrowhead). Scale bars indicate 10μm.

As shown previously by Tritto et al. [26], larval neuroblasts of *pol32* mutants exhibit spontaneous chromosome breaks. We confirmed that result using our *pol32* alleles. From analyzing mitotic chromosome preparations of mutant nuclei, we discovered that 7.7% of the mutant nuclei harbored at least one DSB (n = 766) compared with less than 1% of the wild-type nuclei (n = 2004). We noticed a seemingly non-random distribution of DSBs on mitotic chromosomes of *pol32* mutants. To facilitate the identification of putative “hot spots” for DSB formation, we took advantage of a sensitized background that greatly increases DSB frequency in *pol32* mutants. As described in the next section, we discovered a genetic interaction between components of the Pol δ complex with Pol32. In particular, a heterozygous *polD* mutation exacerbates the phenotypes of *pol32* homozygotes including the frequency of DSB in larval neuroblasts. With this sensitized background, we observed 30.6% of the nuclei having at least one DSB (n = 1375, Fig 7B).

We loosely defined genomic regions on the mitotic chromosomes as “centric” and “non-centric” according to prior cytological studies of mitotic chromosomes in Drosophila [e.g., 35–37]. In brief, the centric domain consists of the centromere constriction, DAPI-bright regions next to the centromere, and the adjacent regions where the sister chromatids remain tightly synapsed. These “centric” regions are generally considered heterochromatin, and the remaining “non-centric” regions are considered gene-rich euchromatin in the genome. Fig 7A shows representative mitotic figures with DSBs in each type of chromatin and on every major chromosome. DSBs of the two different regions were then quantified for each chromosome except the *Y* or the 4^th^ chromosomes (Fig 7B; n = 286 for DSB on *X*; n = 132 for DSB on *II*; n = 179 for DSB on *III*). We discovered that about 70% of the *X* chromosomal DSBs could be defined as “centric”. The frequencies are 42% and 54% for chromosomes *2* and *3* respectively. We did not include the *Y* chromosome in our DSB analyses basing on the rationale that highly condensed heterochromatic regions of the *Y* chromosome might assume the appearance of DSBs [e.g., 38], biasing our quantification. Nevertheless, we did observe mitotic figures showing clearly broken *Y* chromosomes (Fig 7Al). We did not quantify DSBs on chromosome *4* due to its small size and the consequent difficulty in identifying DSBs cytologically. Therefore, we have established an effective way to generate DSBs induced by replication stress and started to define basic features for them.

### The *rDNA* locus is a hot spot for chromosome breakage

*pol32*-mutant adults often express missing, thinning or shortening of large bristles sometimes accompanied by etching of the abdomen (disruption of the normal abdominal pattern owing to cuticular herniations). Some examples are shown in Figs S1 and 8. This is reminiscent of the classic “bobbed” phenotypes caused by loss of copies of the ribosomal RNA gene (*rDNA*) repeats [39], and suggesting that the *rDNA* locus might be experiencing high incidence of instability in *pol32*-mutant cells. The *rDNA* loci reside on the *X* and *Y* chromosomes in Drosophila. We thus carried out a more focused quantification of *X* chromosome DSBs. The effect of *pol32* on the stability of the *rDNA* array on the *Y* chromosome was assayed differently and will be described in a later section.

The major components of the *X* centric region are two large blocks of repetitive sequences: the *rDNA* locus about 3 Mb in size and the more centromere-proximal 359 satellite about 11 Mb in size. We made fluorescent probes to each region and used them in FISH experiments to categorize DSBs on the *X* chromosome. We again used the sensitized background of *pol32* homozygosity with a heterozygous *polD* mutation. Out of 332 nuclei with a complete karyotype and FISH signals, we identified 41 breaks in the *rDNA* locus and 16 DSBs in the 359 repeats. Representative FISH images are shown in Fig 7C. Therefore, under the genetic background in our study, about 1 in 6 (57/332) larval neuroblasts experienced a DSB at the *X* peri-centromeric region.

Because we could now definitively identify some of the DSB sites using FISH, we were able to further characterize the 286 DSBs that we previously identified on the *X* chromosome (Fig 7B) by comparing the patterns of DAPI and FISH signals. We observed two classes of “centric” DSBs on *X*. The first class of DSBs lies in the DAPI bright block (for a representative mitotic figure see Fig 7Af). This class accounts for 20.6% (59/286) of all *X* DSBs. Now FISH analyses clearly show that they happened within the 359 repeats (Fig 7Cd). The second class of DSBs happened in the region right next to the DAPI bright block where the sister chromatids tightly synapse (Fig 7Ad and 7Ae). This class accounts for 49.0% (140/286) of all *X* DSBs. Our FISH data suggest that most, if not all, of this class of DSBs represent breaks of the *rDNA* locus (Fig 7Cb and 7Cc). Therefore, almost half of *X* breaks were at *rDNA*. In addition to abundant DSBs involving *rDNA*, we also observed instances in which the broken ends of the *rDNA* locus joined with other broken ends giving rise to genome rearrangements (Fig 7Ce), and instances in which the *rDNA* array appears expanded (Fig 7Cf).

To further quantify the damage to the *rDNA* loci and to prove that *rDNA* instability is not limited to the array on the *X* chromosome, we made the *Y* chromosome the sole source of *rDNA* in *pol32* mutants by introducing the *pol32* mutation into a *C(1)DX*, *rDNA*^*0*^*/y*^*+*^*Y10B* background. The former chromosome (compound *X*) lacks all *rDNA* and the latter (*Y*) possesses a well-characterized *rDNA* array [40]. We extracted genomic DNA from females (*C(1)DX/Y*) of both *pol32* mutants and *pol32/+* siblings. The *pol32* mutants had 57.0% (±11.4%) the rDNA copy number as did their heterozygous siblings. This amount (about 60% of the normal level) roughly corresponds to the threshold between the extreme-bobbed/bobbed-lethal boundary, suggesting that the surviving *pol32* flies have as little *rDNA* as can sustain development. Despite the overall loss of *rDNA*, when compared to total rDNA copy number, mutants had 3.2 times as many *rDNA*-resident R1 retrotransposons and 1.5 times as many R2 retrotransposons as did their heterozygous siblings. The R1 and R2 elements are generally kept silent in the *rDNA* arrays [41, 42], and we suggest that the preferential loss of *rDNA* copies uninterrupted by R1 or R2 indicates that the loss due to the *pol32* mutations involves active *rDNA* arrays. Therefore, in a replication compromised background, both of the *rDNA* loci experience a high rate of instability.

### Chromosome breakage in *pol32* mutant is primarily due to defects in replication

Although it is likely that defective replication is the primary cause for the spontaneous DSBs that we observed in the mutants, it is possible that another significant cause is the loss of repair capacity as Pol δ is important in DSB repair [e.g., 43]. To shed more light onto the primary cause(s), we conducted a genetic interaction study of *pol32* with mutations in other replication and DNA repair factors.

**Fig 8:**
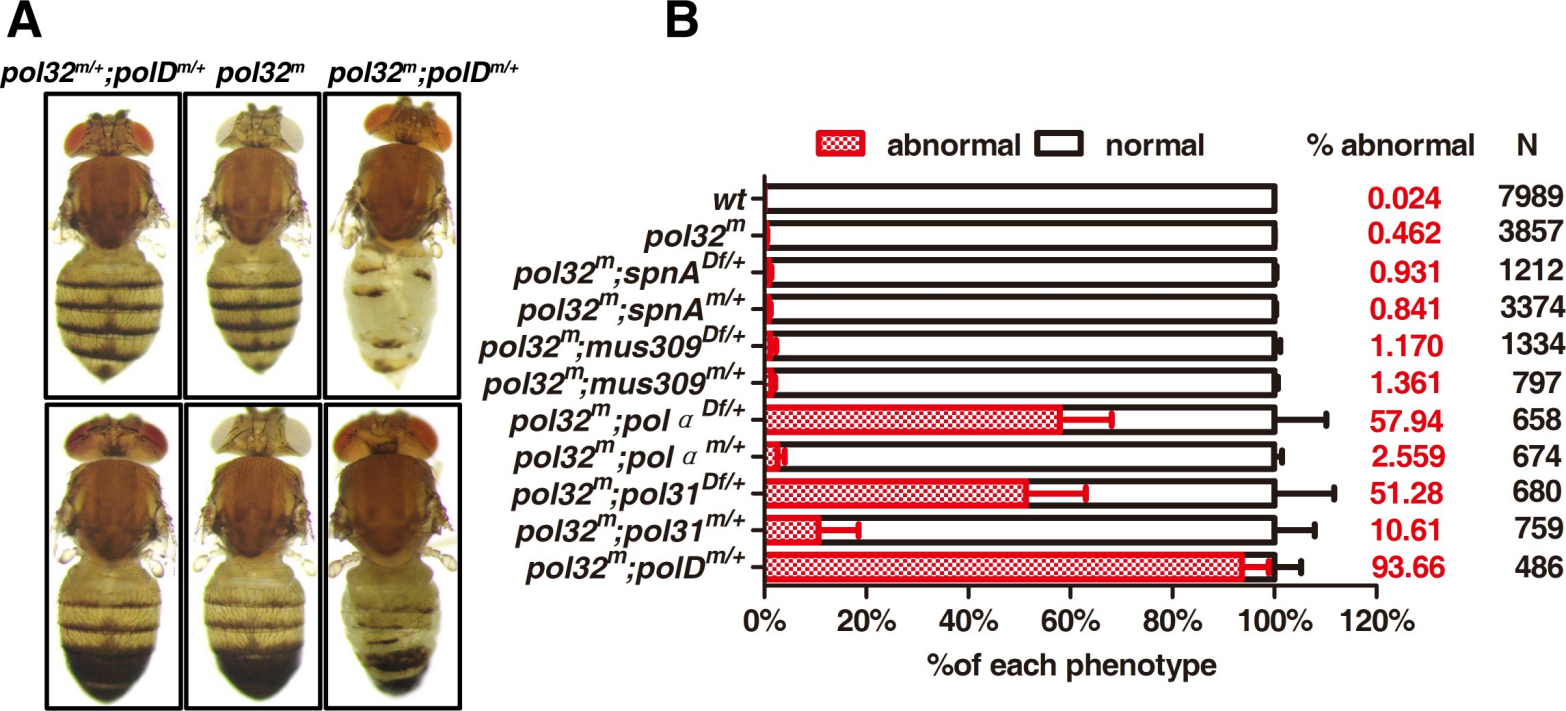
Genetic interactions between *pol32* and other DNA replication and repair factors. **A**. Pictures of adult flies (three females at the top and three males at the bottom) showing etching of the abdominal region. Genotypes are given at the top. **B**. Quantification of the “etched abdomen” phenotype. The specific mutant alleles (*m*) are as followed: *pol32*^*m*^ *= pol32*^*L27/L30*^, *spnA*^*m*^ *= spnA*^*1*^, *spnA*^*Df*^ *= Df(3R)X3F*, *mus309*^*m*^ = *mus309*^*D2*^, *mus309*^*Df*^ = *Df(3R)P-21*, *pol*α^*m*^ *= pol*α^*G13925*^, *pold*^*m*^ *= pold*^*l10*^, *pol*α^*Df*^ = *Df(3R)Exel6186*, *pol31*^*m*^ *= pol31*^*G16501*^, *pol31*^*Df*^ = *Df(3L)Bsc122*.

Our assay was based on the etching of the abdominal region of *pol32* adults (Fig 8A). We observed *pol32* homozygotes with etched abdomen at a low frequency: 0.5% of the adults displayed the phenotype. This was increased to 93.7% when a copy of the *polD* gene was also mutated (homozygous for *pol32* but heterozygous for *polD*), and 51.3% when we deleted a copy of the *pol31* gene (homozygous for *pol32* but heterozygous for *pol31*). Interestingly the strength of the genetic interactions between *pol32* and *pol31* appears proportional to the strength of the heterozygous mutations, as a hypomorphic *pol31* mutation (a homozygous viable mutation with a *P* element insertion at the 5’UTR of *pol31*) had a weaker enhancing effect (from 0.5% to 10.6%) than a complete deletion of *pol31* (from 0.5% to 51.3%). These results indicate a strong genetic interaction between Pol δ subunits. Using the same assay, we tested mutations in *pol*α, and observed a similar enhancement. Interestingly, when we tested two other factors with important roles in DNA repair, *mus309* encoding the Drosophila homolog for the Bloom RecQ helicase [44] and *spnA* encoding the Drosophila homolog for the Rad51 strand annealing protein [45], we obtained weaker enhancement of the *pol32* phenotype (Fig 8B). Alleles used for *mus309* and *spnA* were previously shown to be strong, if not complete, loss of function alleles [44, 46].

As shown in the previous section, the strong interaction between *pol32* and *polD* reflects well the frequency of spontaneous DSBs such that the *polD* mutation also enhances DSB formation frequency in *pol32* homozygotes (Fig 7B). Therefore, our cytological and genetic results combined suggest that DSB formation in a *pol32* mutant background is largely due to a defect in genomic replication, and less so DSB repair.

## Discussion

Here we presented a developmental study on the function of Pol32, an important subunit of DNA polymerase δ. By characterizing *pol32* function in different cell types, we identified a previously underappreciated function of Pol32 as a facilitator of the nuclear import of Pol δ. This opens up a new front for the study of polymerase ancillary factors in eukaryotes. In addition, we established an effective way to generate spontaneous chromosomal breaks by causing non-lethal disruption of genomic replication. This paves the way for future studies aimed at characterizing the fundamental features of chromosomal fragile sites in eukaryotes.

### Pol32 is required for efficient nuclear localization of the polymerase complex

We discovered that although Pol32 is not essential for organismal survival, the maternal pool of Pol32 protein is absolutely required for early embryonic development. The early embryonic arrest phenotype is associated with a severe defect in whole genome replication so that as much as 90% of the total embryonic DNA present is of mitochondrial origin. Analysis of our sequencing data did not identify specific regions of the genome that are less represented in mutants, suggesting that the disruption of replication is genome-wide. This is the most severe replication defect ever reported for *pol32* mutants in any system. The cessation of replication is associated with a severe disruption of the localization of PolD and Pol31 proteins, the remaining subunits of the polymerase complex. This nuclear localization defect occurs during the earliest zygotic DNA replication, was not the result of protein instability, and is specific to the Pol δ complex. Therefore, Pol32 is required for Pol δ localization during early embryonic development.

In post-embryonic cells, however, this requirement is much less stringent. Although *pol32*-mutant cells show spontaneous DSBs, a consequence of sub-optimal replication function, they are nevertheless proficient in tissue proliferation suggesting that the Pol32-less complex retains substantial function including the ability to enter the nucleus, a proposition supported by our immunostaining results. As we discuss below that there might be redundant mechanisms controlling the nuclear transport of PolD, a systematic approach is therefore required to determine whether Pol32 is at all required for the normal nuclear localization of Pol δ in post-embryonic cells. It is also of great interest to further understand the striking differences displayed between embryonic and post-embryonic cells in response to the loss of Pol32.

### Functional similarity between Pol32 and accessory factors of viral polymerases in mammals

**Fig 9:**
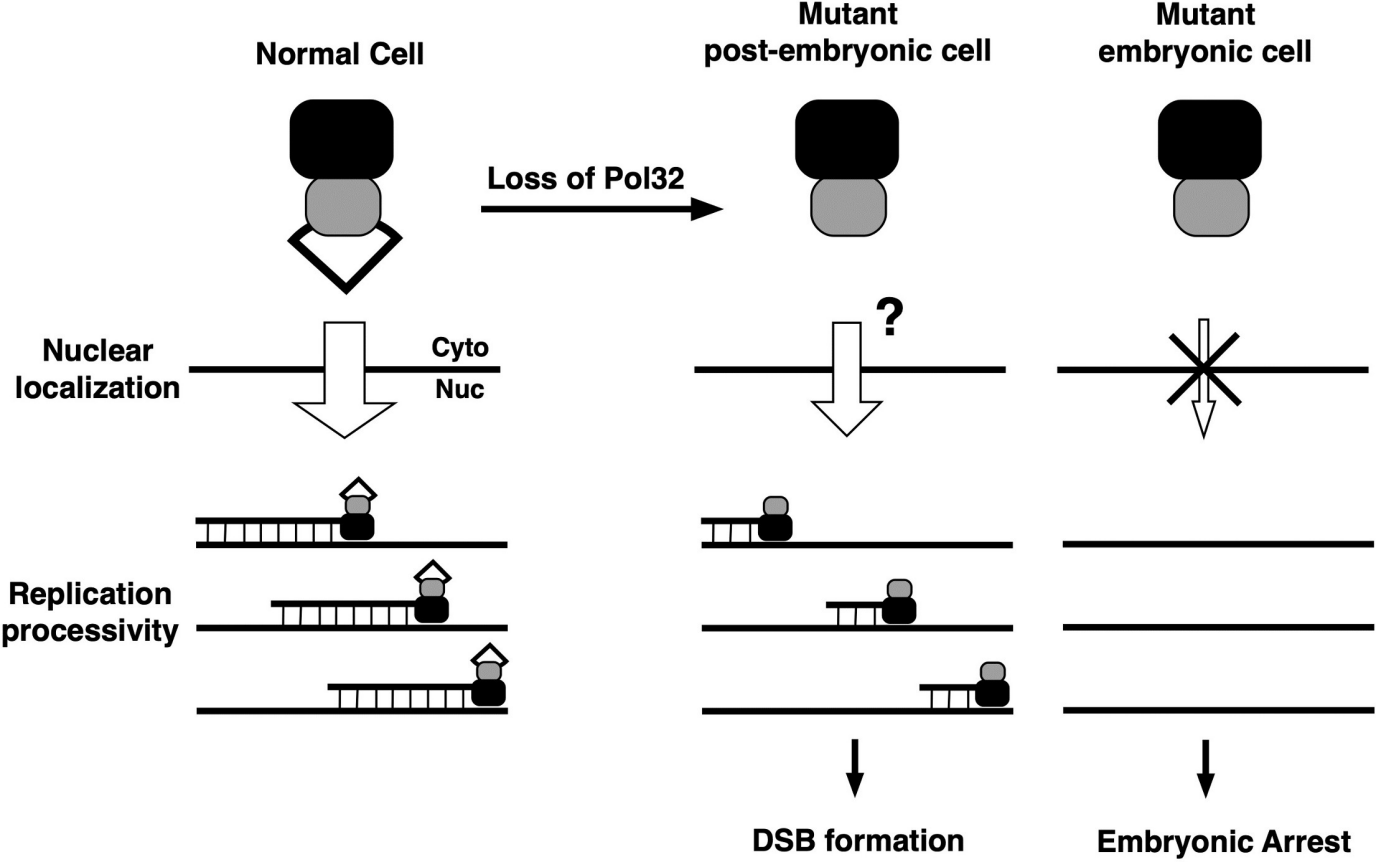
A speculative model for the dual function of Drosophila Pol32. The normal Pol δ complex consists of PolD (black box), Pol31 (grey box) and Pol32 (white triangle). In normal cells, the cytoplasmically assembled Pol δ enters the nucleus efficiently, and possesses normal polymerase processivity leading to efficient DNA synthesis (long strands of newly synthesized DNA). In *pol32*-mutant post-embryonic cells, Pol δ might enter the nucleus at a reduced efficiency (smaller arrow and question mark), accompanied by the partial loss of processivity (shortened strands of new DNA). This leads to the formation of chromosome breaks. In *pol32*-mutant embryonic cells, however, Pol δ is inhibited from entering the nucleus halting genome replication and embryonic development.

We have devised a speculative model for Pol32 functions (Fig 9). We propose that Pol32 fulfills two separable functions: a factor facilitating the nuclear localization of Pol δ, and a processivity factor for efficient catalytic activity of Pol δ. This proposed dual function of Pol32 is similar to those assigned to viral replication factors from certain mammalian viruses.

The genomes of viruses of the Herpesvirdae family, and similar viruses of other mammalian hosts, are replicated by a set of viral factors that include a two-subunit DNA polymerase. In this complex, the polymerase catalytic subunit is accompanied by an ancillary factor, such as the UL42 protein of the HSV-1 virus, UL44 of HCMV, BMRF1 of EBV and ORF59 of KSHV [for a review on viral polymerases see 47]. The ancillary factor greatly increases the processivity of the catalytic enzyme *in vitro* [e.g., 48, 49], similar to results from studying Pol32. Interestingly, these viral replication processivity factors also regulate and sometimes control the nuclear import of the polymerase complex [reviewed in 50]. The processivity factors can be classified into two classes in terms of how they participate in the nuclear import of the polymerase complex. In one class, exemplified by UL42 and UL44, the processivity factor as well as the catalytic subunit harbor functional nuclear localization signals. Each mediates nuclear import of the complex in a redundant fashion [51–53]. In the other class, exemplified by BMRF1 and ORF59, only the processivity factor has a functional nuclear localization signal(s) and is solely responsible for importing the entire complex into the nucleus [54–56]. Therefore, Pol32 is similar to the first class of viral processivity factors, as the Pol δ complex is capable of entering the nucleus without Pol32 in single cell systems such as yeast, chicken cells, cultured mammalian cells, and in post-embryonic cells of mice and flies. The striking exception is early embryonic cells of Drosophila, and possibly mouse, in which Pol32 is vitally required for nuclear import. This might reflect the specialized replication program in early divisions. Interestingly, partial loss of function mutations in other Drosophila replication factors can cause maternal effect embryonic lethality similar to *pol32* [e.g. 57].

The viral processivity factor mediates nuclear import via conventional importin-based mechanisms [e.g., 51, 52, 58]. This might also be the case for Pol32 function. It would be even more interesting that the stringent requirement for Pol32 in embryos be based on cell-type specific interaction between Pol32 and the nuclear import machinery. A functional nuclear localization signal (NLS) has been identified for human p66 [59], and we identify a putative NLS in Drosophila Pol32 based on the mammalian finding. We predict that the disruption of this NLS would reproduce the embryonic phenotypes similar to that caused by the current *pol32* mutations.

The model in Fig 9 also indicates that the processivity function of Pol32, possibly combined with a defect in Pol δ nuclear import, can explain the appearance of DSBs in mutant somatic cells. There is likely contribution from defects in DNA repair in the mutant as Pol32 is also a subunit of DNA polymerase ζ [11, 13], which is essential for DNA repair in Drosophila [25].

### Repetitive sequences are not necessarily chromosomal fragile sites in Drosophila

Chromosomal fragile sites (CFS), also called Common Fragile Sites, represent genomic regions with the propensity to break under replication stress [for a recent review of CFS see 60]. CFS are best studied in mammalian systems in which “difficult-to-replicate” regions of repetitive sequences capable of forming secondary structures and regions with the likelihood of collisions between replication and transcription machineries are CFSs [reviewed in 61–63]. We established a genetic background in which a cell’s replication efficiency was reduced so that 30% of proliferating cells possess at least one chromosome break. Remarkably, animals of such genetic makeup survive to adulthood. With this abundance of DSBs, we were able to detect some interesting features about the classes of DSB that arose as a result of replication inefficiency.

We classified CFS into centric and non-centric classes. The regions that we defined as “centric” fit the general description of heterochromatin on mitotic chromosomes [35, 37, 64]. Approximately one-third of the Drosophila genome can be classified as “heterochromatic” [65, 66]. Yet centric DSBs that we observed account for 55.4% of the total DSBs. Therefore, heterochromatic regions in Drosophila seem to have a higher propensity to express CFS. However, an alternative classification of the chromatin state at the *rDNA* locus could significantly reduce the over-representation of heterochromatic DSBs.

Although the *rDNA* locus in Drosophila is generally considered heterochromatic [65], it can show different staining patterns from classical heterochromatic regions with dyes commonly used to define heterochromatin [e.g., 36]. In addition, the *rDNA* locus, although being repetitive, is one of the most expressed loci in the genome. If we were to take DSBs in *rDNA* out of consideration as DSBs in heterochromatin, we would reach a new estimate of 28% as heterochromatic DSBs, a number closer to 33%, the estimated heterochromatic proportion of the genome. Therefore, we suggest that the occurrence of DSBs induced by replication stresses does not favor the transcriptionally silent heterochromatic regions. In other words, being repetitive does not necessarily render a region more susceptible to breakage. This proposition is further supported by DSB frequency that we observed for the 359 satellite. The satellite is 11 Mb in size, about 30% of the *X* chromosome, consisting of tandem repeats of a 359bp element [67]. We observed that only 20.6% of the *X* DSBs happened within the satellite. Even if we were to take *rDNA* breaks (49% of *X* breaks for about 9% of the size of *X*) out of the calculation, the 359 satellite would account for about 35% of the size of the remaining *X* and 40% of the DSBs on the remaining *X*. Therefore, our analyses both genome-wide and of the specific 359 satellite locus support our proposition on the lack of a correlation between sequence repetitiveness and CFS expressivity.

The *rDNA* locus is one of the most studied loci for replication induced instability. It has been shown in multiple studies that *rDNA* is highly sensitive to replication deficiency [e.g., 68, 69]. Our study confirmed that this general rule also applies to Drosophila *rDNA*. In larval neuroblasts under a replication-compromised background, one in eight cells suffers a DSB at *rDNA*, and half of the DSBs on *X* happens at *rDNA*. The likely cause for this high rate of DSB formation is the collision between DNA replication and transcription machineries, which is consistent with our results showing the preferential loss of active *rDNA* cistrons from the arrays on the *Y* chromosome. This form of replication stress mechanism has been well studied before [e.g., 70].

### The future of CFS studies in Drosophila

Although we have developed a condition to induce high rates of DSBs in somatic cells, to reveal common features of these fragile sites requires an efficient way to identify the broken region. This is particularly important for DSBs happened in the euchromatic regions. Preferably, a genetic method can be devised to isolate and propagate the chromosomes with the broken end so that further cytological and molecular characterizations of these ends could be carried out. This has been challenging for the mammalian systems since such broken ends are most often lost due to its inability to acquire a functional telomere. In contrast, such isolation is feasible in Drosophila, an organism that naturally lacks the telomerase enzyme and essentially any sequence can be a part of a functional telomere [for a review see 71]. We and others have shown that broken chromosomes can be effectively “healed” in the germline [e.g., 72, 73] and an elegant scheme of isolating broken ends of a ring-*X* chromosome in the germline has been successfully implemented [74], which will greatly facilitate our future efforts in systematically isolating and characterizing CFS in Drosophila.

## Materials and methods

### Drosophila stocks

Drosophila stocks were raised on cornmeal medium under standard laboratory conditions. The *mus309*^*D2*^ stock was a gift from Dr. Jeff Sekelsky at UNC. Other stocks were obtained from the Bloomington *Drosophila* stock center and described in FlyBase (flybase.net), with the stock numbers shown below in parentheses: *spnA*^*1*^ (3322); *pol31*^*G16501*^ (27423); *pol31* deficiency (9142); *polα*^*G13925*^ (31805); *polα* deficiency (7665); *polD*^*l10*^ (4110); *mus309* deficiency (6167); *spnA* deficiency (2352). The two stocks carrying RNAi hairpins (Fig 6C) against *pol31* were obtained from Vienna Drosophila Resource Center with the stock numbers of V108565 (hairpin #1) and V13621 (hairpin #2). They were driven by a *tubulin-Gal4* gene. The estimation of *rDNA* copy numbers was performed as previously described [40, 42].

### Generating mutations and transgenes

Two mutant alleles of *pol32* (L27 and L30) were recovered by mobilizing *P* element *P{EPgy2}pol32*^*EY15283*^ from the 3’ region of *pol32*. For *pol32*^*L27*^, nt15255461 to nt15256304 were deleted (nt designations are based on FlyBase version FB2018_05) with an additional 162bp of filler sequences from the *P* element. For *pol32*^*L30*^, nt15255502 to nt15256304 were deleted with an addition of 39bp of *P* element sequences. These two alleles express identical phenotypes. Since only part of the *pol32* coding region was removed in each of the mutant alleles (along with the entire annotated *3’UTR*), there is still a possibility that a truncated Pol32 protein with 205 (*L27*) or 219 (*L30*) Pol32 residues was produced by the mutant genes even though the truncated protein were undetectable by Western blot analyses.

To construct a rescuing transgene for *pol32* mutants, a 4kb fragment (nt15254079 to nt15257317, FB2018_05) was PCR-amplified from wild-type genomic DNA. The DNA was confirmed by sequencing and cloned into pUAST-attB for phiC31-mediated germline transformation of Drosophila [75]. To generate transgenes with various point mutations or domain deletions, site-specific mutagenesis was performed on the pUAST-attB construct with the wild-type *pol32* fragment, followed by verification of the mutations by sequencing. Two independent lines for each transgene construct were used to rescue *pol32*^*L30*^ homozygous flies.

### Genome sequencing

Embryos (0-2h after egg-laying) from *pol32*^*L30*^ and *w*^*1118*^ females were collected and genomic DNA was extracted by standard methods. A total of 1.5μg DNA per sample was used for whole genome sequencing performed by Novogene (Guangzhou, China) using the Illumina HiSeq platform. Reads were mapped to the reference genome (*Drosophila melanogaster* Release 6 plus ISO1 MT).

### Antibodies

Guinea pig anti-Pol32 antibodies were raised against the full length Pol32 protein purified as a recombinant protein from *E*. *coli*, and affinity-purified using the same antigen. The Pol32 antibodies were used at 1:5000 on Western blots and1:1000 in immunostaining experiments. Mouse anti-PolD (CG5949) and anti-Pol31 (CG12018) sera were raised against the first 238 a.a. (PolD) and the full-length protein (Pol31) as antigens purified from bacteria, and used at 1:5000 and 1:1000 on Western blots, and1:1000 and 1:100 in immunostaining experiments, respectively. Mouse anti-Polα (CG6349) sera were raised against a recombinant antigen consisting of residues 411–705, and used at 1:1000 on Western blots and 1:100 in immunostaining experiments. Mouse anti-α-tubulin (Sigma, DM1A) was used at 1:10000 on western blots. Mouse anti-PCNA (Abcam, ab29) was used at 1:5000 on Western blots and 1:1000 in immunostaining experiments.

### Co-immunoprecipitation

Embryos collected every 2 hours were homogenized in IP binding buffer (PBS supplemented with 0.3% Triton X-100 plus protease inhibitor cocktail tablets from Roche). An anti-PolD serum (5μl) or a purified anti-Pol32 antibody (5μl) or an anti-Pol31 serum (5μl) was added to the embryonic extracts and incubated for 3h at 4°C. Protein A/G agarose from Santa Cruz (20μl for each sample) was added to the above mixture and incubated for 1h at 4°C. The beads were washed 3 times each with 1ml of IP binding buffer. Bound protein complexes were eluted with SDS sample buffer, and resolved by SDS–PAGE for Western blot analysis.

### Immunostaining

Adult ovaries were dissected in fresh PBS and fixed with freshly diluted 3.7% formaldehyde in PBS for 20 min at room temperature. Subsequent immunostaining was performed with a standard protocol. For embryo staining, embryos were collected every 15min and dechorionated with 50% bleach and washed with embryo wash buffer (0.7% NaCl, 0.02%Triton X-100), then fixed with 1:1 freshly diluted 3.7% formaldehyde in PBS and heptane. Subsequent immunostaining was performed with a standard protocol. Fluorescent images were taken with an Olympus IX83 confocal microscope.

### Mitotic chromosome squash

Mitotic chromosome squash of neuroblasts from third instar larvae were prepared following a standard protocol without a colchicine treatment. Chromosome preparations were analyzed with a Zeiss Axio Image A2 microscope.

### Fluorescent *in situ* hybridization (FISH) on mitotic chromosomes

Brains were dissected from wandering third instar larvae and squashed in 45% acetic acid. The slides were fixed in freshly made 4% formaldehyde in PBS for 20 min at room temperature and washed twice with 2×SSC. Slides were dehydrated in 70% ethanol for 10 min twice then in 95% ethanol for 5 min, followed by air drying. Hybridization was performed in 50% formamide, 10% dextran sulfate, 2×SSC, 0.5μM of each probe and up to 20μl of dH_2_O. The slides, covered with a coverslip, were heated to 91°C for 2 min, cooled briefly and incubated in a humid chamber in the dark overnight at room temperature. Post-hybridization washes were done three times in 0.1×SSC for 15 min each, and the slides were stained with DAPI (0.2μg/ml in 2×SSC) for 5 min, washed briefly in 2×SSC and allowed to air dry. Slides were mounted in Vectashield from Thermo and analyzed with a Zeiss Axio Image A2 microscope. The sequences of fluorescent probes from the 359 satellite and the *rDNA* IGS were the same as ones used by Jagannathan et al. [76].

## Supporting information

S1 Fig Bristle phenotype of *pol32* adults. Pictures of a *pol32* homozygous adult (white-eyed) and similar adults (red-eyed) with a rescuing construct described in Fig 5A. The presence of the *white*^*+*^ gene in the rescuing construct gives rise to eye color. Instances are marked with arrowheads where the largest bristles are missing.
(TIF)

S2 Fig The specificity of the Pol32 antibody. In **A**, total extracts from wild-type and mutant ovaries were used on a Western blot with the position of the Pol32 band and the sizes of the protein markers indicated. Tubulin was used as a loading control. In **B**, mutant ovaries were stained with anti-Pol32. A separate image is provided for the DAPI signal (in white), the anti-Pol32 signal (in red), and the merged product of the two channels. For the antibody channel, the images were overexposed to show the general lack of Pol32 in the nucleus. In the three panels of enlarged images to the right, **Ba** represents nuclei from nurse cells. In **Bb**, the chromosomes in the oocyte nucleus are marked with an arrowhead. **Bc** represents nuclei from follicle cells. Scale bars in red indicate 40μm, and 10μm in white.
(TIF)

S3 Fig Pol δ localization is regulated in early embryonic cycles. Embryos (0-2hr after egg laying) were used in immunostaining experiments. Each group of images consists of one showing interphase nuclei (top) and one showing metaphase nuclei (bottom), with DAPI and two antibody staining images and the merged product of the three. Scale bars indicate 10μm.
(TIF)
