# Supplementary figures and images for: The processivity factor Pol32 mediates nuclear localization of DNA polymerase delta and prevents chromosomal fragile site formation in Drosophila development
Source: PLoS Genet. 2019 May 17;15(5):e1008169. doi: 10.1371/journal.pgen.1008169 (PMC6542543; doi:10.1371/journal.pgen.1008169)

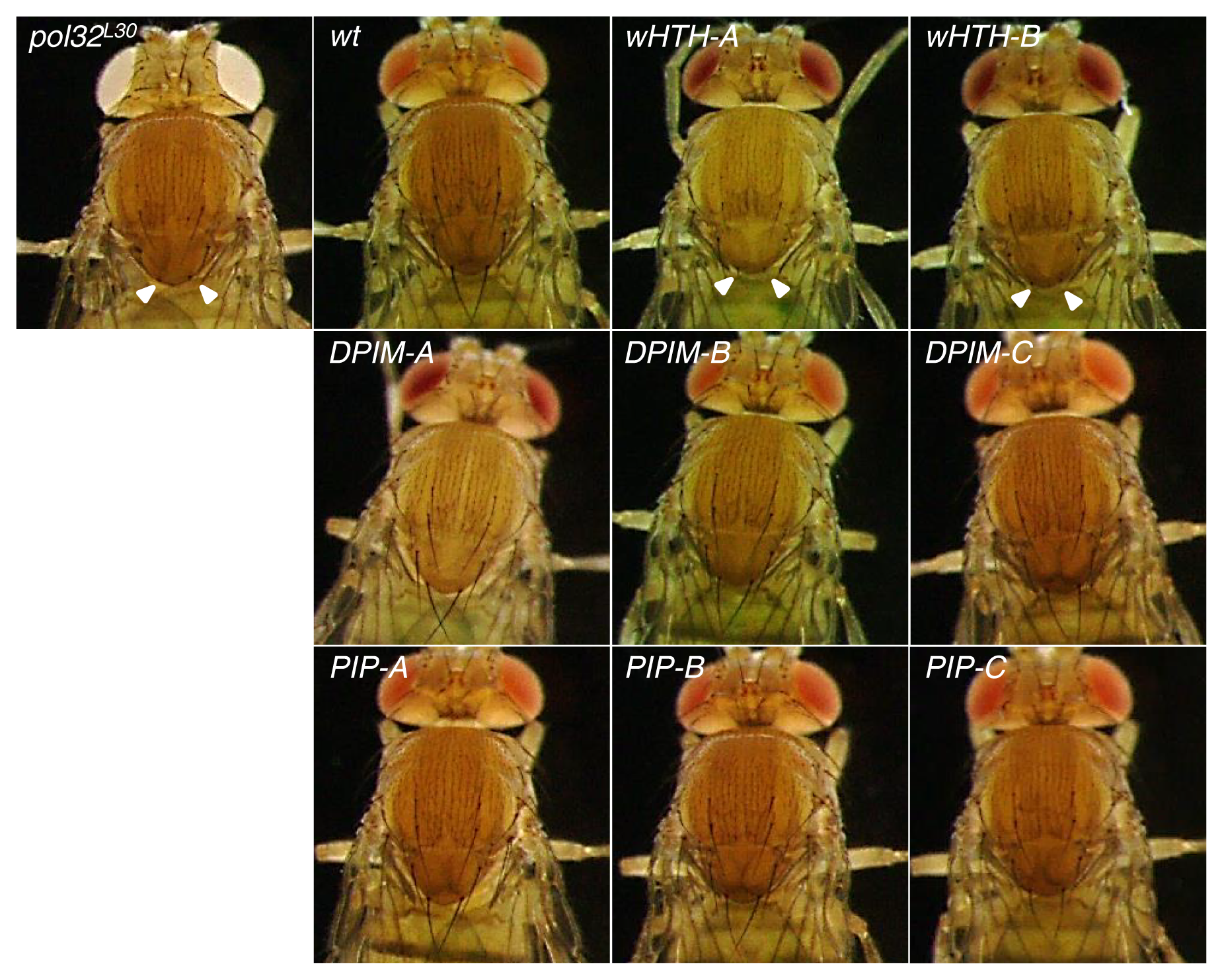

Supplement: S1 Fig — Pictures of a pol32 homozygous adult (white-eyed) and similar adults (red-eyed) with a rescuing construct described in Fig 5A. The presence of the white+ gene in the rescuing construct gives rise to eye color. Instances are marked with arrowheads where the largest bristles are missing. (TIF) [file pgen.1008169.s001.tif]

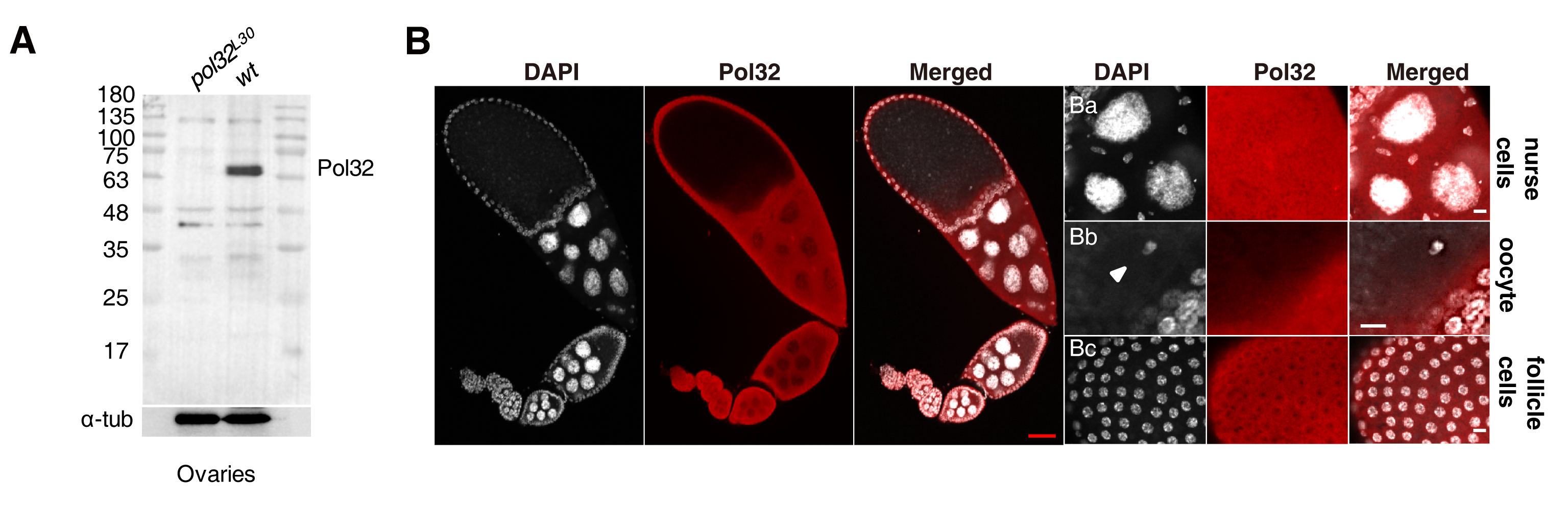

Supplement: S2 Fig — In A, total extracts from wild-type and mutant ovaries were used on a Western blot with the position of the Pol32 band and the sizes of the protein markers indicated. Tubulin was used as a loading control. In B, mutant ovaries were stained with anti-Pol32. A separate image is provided for the DAPI signal (in white), the anti-Pol32 signal (in red), and the merged product of the two channels. For the antibody channel, the images were overexposed to show the general lack of Pol32 in the nucleus. In the three panels of enlarged images to the right, Ba represents nuclei from nurse cells. In Bb, the chromosomes in the oocyte nucleus are marked with an arrowhead. Bc represents nuclei from follicle cells. Scale bars in red indicate 40μm, and 10μm in white. (TIF) [file pgen.1008169.s002.tif]

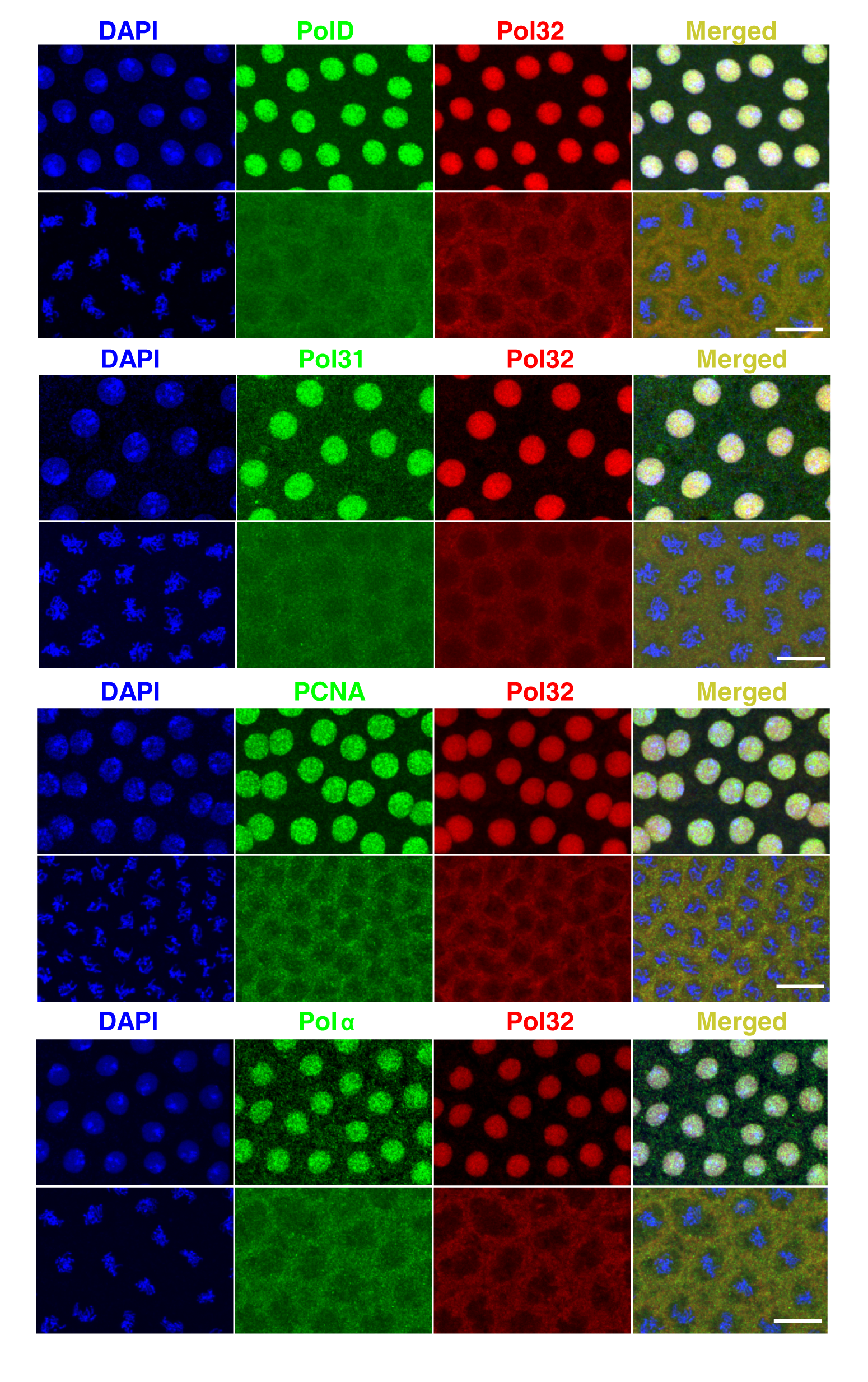

Supplement: S3 Fig — Embryos (0-2hr after egg laying) were used in immunostaining experiments. Each group of images consists of one showing interphase nuclei (top) and one showing metaphase nuclei (bottom), with DAPI and two antibody staining images and the merged product of the three. Scale bars indicate 10μm. (TIF) [file pgen.1008169.s003.tif]
